# Choroidal Recurrence of Testicular Diffuse Large B‐Cell Lymphoma: A Diagnostic and Therapeutic Challenge in an Immune‐Privileged Site

**DOI:** 10.1155/carm/7243012

**Published:** 2025-12-22

**Authors:** Veysel Erol, Osman Parça, Nevin Alayvaz Aslan, Nilay Şen Türk

**Affiliations:** ^1^ Department of Hematology, Faculty of Medicine, Pamukkale University, Denizli, Turkey, pau.edu.tr; ^2^ Department of Ophthalmology, Faculty of Medicine, Pamukkale University, Denizli, Turkey, pau.edu.tr; ^3^ Department of Pathology, Faculty of Medicine, Pamukkale University, Denizli, Turkey, pau.edu.tr

**Keywords:** central nervous system lymphoma, choroidal lymphoma, immune-privileged site lymphoma, testicular lymphoma

## Abstract

The recent inclusion of immune‐privileged site lymphoma in the World Health Organization classification signifies a distinctive entity encompassing the central nervous system and testicles. This classification is rooted in the unique challenges posed by the blood–brain barrier and blood–testis barrier, along with the distinct cancer microenvironment compared to standard lymphomas. These intricacies contribute to the complexity of treatment strategies, and as of now, a standardized protocol remains elusive. Our presented case underscores the critical need for a comprehensive treatment plan in immune‐privileged site lymphomas, where surgery alone, in the form of indolent intervention, may fall short in addressing the underlying aggressive nature of the disease. Failure to administer systemic treatment, where indicated, heightens the risk of aggressive recurrence and substantially elevates mortality rates. Through a detailed examination of our case, we aim to contribute to the evolving body of knowledge surrounding these unique lymphomas, offering valuable insights that may guide future treatment strategies and improve patient outcomes.

## 1. Introduction

Immune‐privileged site (IPS) is a new concept and refers to the region where an antigen, which will be detected and eliminated by immune mechanisms under normal conditions, cannot be noticed by the immune system and can survive in the tissue for a long time. The central nervous system (CNS) and testes are good examples to these [1].

The treatment and prognosis of CNS and testicular lymphoma differ from other lymphomas. Factors such as the presence of blood–brain and blood–testis barriers, lack of antigen‐presenting cells and lymphatic drainage, and different tumor microenvironment structures, compared to other lymphomas, are the most important causes of resistance to treatment, early relapse, and poor prognosis [2, 3]. Primary CNS lymphoma is seen in 2%–4% of all lymphomas [4]. CNS lymphoma includes brain parenchyma, spinal cord, leptomeninges, and intraocular region lymphomas (IOLs). IOL constitutes only 1% of all non‐Hodgkin’s lymphomas [5]. IOL is also categorized by the involvement of uveal and vitreoretinal regions. While uveal region involvements are associated with iris, ciliary body, and choroidal involvement and progress more indolently, vitreoretinal region involvements are more aggressive due to their frequent association with CNS involvement. Choroidal lymphoma mostly occurs following CNS metastasis of systemic lymphoma [6]. When choroidal lymphoma is considered on its own, it is mostly associated with indolent lymphomas, such as mucosa‐associated lymphoma in its primary involvement, and secondary choroidal lymphoma forms are mostly associated with lymphomas with an aggressive course [7]. Although there are few reviews over the past 2 decades, reviews consistently underscore a critical concern: the diagnosis of this condition often faces delays of 2–3 years, leading to diminished long‐term survival rates. Compounding this issue, there is no universally accepted treatment protocol, exacerbating the challenge of managing the condition effectively. Moreover, approximately half of cases suffer from inadequate tissue diagnosis, largely due to the thin structure of the choroid [7–9].

Although the prevalence of testicular lymphoma is 1%‐2% among all lymphomas, it is the most common testicular malignancy in male patients over 60 years of age [10]. Contralateral testis and CNS recurrence is common after treatment. They are mostly of nongerminal origin and CNS recurrences have a poor prognosis [11, 12]. In recent studies, 5‐year life expectancy has been reported as 66%–85% [13].

These cases are difficult to manage due to the rarity of choroidal lymphoma and testicular lymphoma, mostly aggressive nature of these lymphomas, such as diffuse large B‐cell non‐Hodgkin’s lymphoma (DLBCNHL), and the lack of standard treatment protocols for IPS lymphomas. In this report, we present the treatment of our patient, who could not be followed up for 18 months after the diagnosis of testicular lymphoma was deemed as recurrence with isolated choroidal metastasis without tissue diagnosis.

## 2. Case Presentation

**Figure 1:**
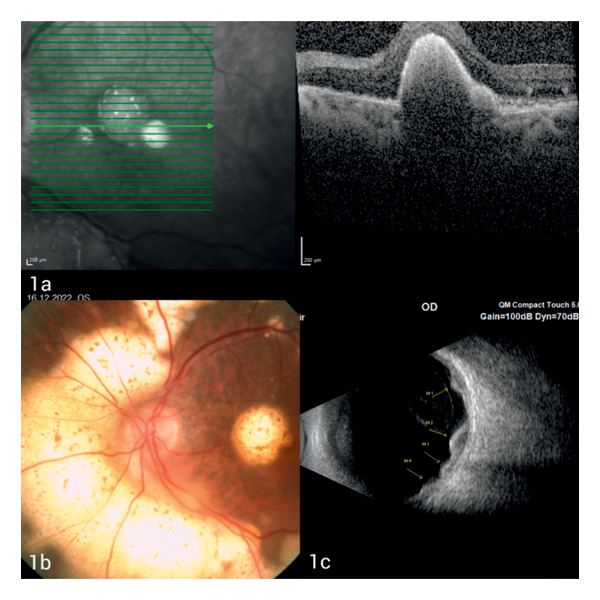
(a) Macular OCT of the left eye demonstrated large elevated subretinal infiltrates. (b) Dilated fundus examination revealed diffuse yellowish subretinal infiltrates around the optic disc and macular region. (c) Ophthalmic ultrasound showed subretinal mass lesions diffusely distributed in a scattered pattern.

**Figure 2:**
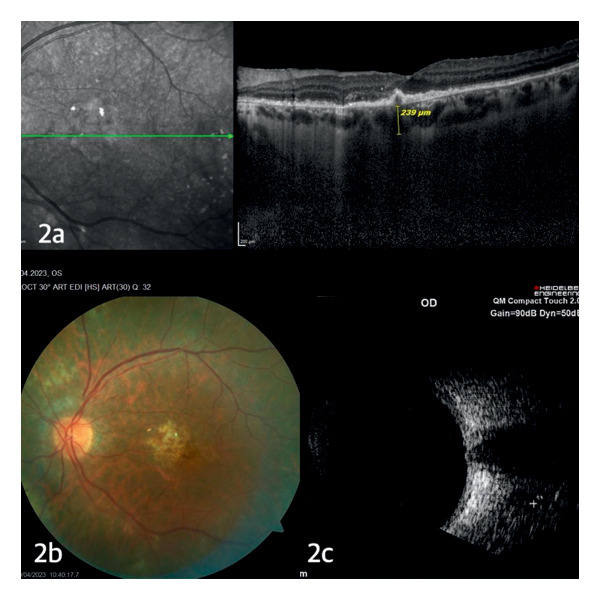
(a–c) Fundoscopic, OCT, and ultrasonographic evaluation after the fourth dose of high‐dose methotrexate treatment showed significant reduction of the choroidal infiltration.

A 72‐year‐old male patient was referred to the ophthalmology department due to sudden loss of vision in the left eye. In the anamnesis of the patient, it was learned that the patient had undergone right orchiectomy due to scrotal swelling 18 months ago and as a result of pathological examination, he had been diagnosed with germinal center type DLBCNHL, but the patient did not comply with follow‐up after the diagnosis since he had been feeling well. In the immunohistochemical examination of the patient’s testicular biopsy, neoplastic lymphoid cells CD20, CD19, CD10, MUM‐1, Bcl‐2, and CD5 (+) and CD3, Bcl‐6, C‐MYC, Cyclin‐D1, TdT, CD138, and CD30 (−) were detected. Ki‐67 proliferation index in neoplastic lymphoid cells was found to be around 30%–50%. The patient had no additional diseases, except early stage prostate adenocarcinoma and coronary artery disease. He had not undergone any additional operations other than coronary artery bypass surgery, prostatectomy, and orchiectomy. At the time of admission, ophthalmologic evaluation revealed visual acuity limited to hand motion in the left eye, while the right eye had a preserved best‐corrected visual acuity (BCVA) of 1.0. Right eye examination was normal. In the slit‐lamp examination of the left eye, no inflammatory cells were detected in the anterior chamber, but +1 cells were detected in the posterior vitreous. Macular OCT of the left eye demonstrated large elevated subretinal infiltrates (Figure 1(a)) and dilated fundus examination revealed diffuse yellowish subretinal infiltrates around the optic disc and in the macular region (Figure 1(b)), and ophthalmic ultrasound showed subretinal mass lesions diffusely distributed in a scattered pattern (Figure 1(c)). On the basis of these findings, the patient was considered to have choroidal metastasis and was referred to our hematology clinic. With the preliminary diagnosis of metastasis of possible systemic lymphoma, PET‐CT and brain and orbital MR scans were taken, but no other lymphoma involvement was detected in the CNS and peripheral lymph nodes of the patient. Complete blood count and biochemical tests were within normal limits. In scrotal ultrasonography (USG), no pathology was detected except for a simple cyst in the left testis. In the bone marrow biopsy, no infiltration in favor of lymphoma was detected. Lumbar puncture cytology and flow cytometry samples were also reported as normal. Since the pathology sample at the time of diagnosis was compatible with aggressive lymphoma and there was no other peripheral involvement in the past 18 months, a second center’ s opinion was requested for testicular biopsy sample to confirm diagnosis and the pathology report was the same with respect to the tissue diagnosis in the primary center’s evaluation. Transvitreal fine‐needle aspiration biopsy (TFNABx) was recommended to the patient, who did not have lymphoma involvement elsewhere, but the patient did not accept the TFNABx procedure due to risks such as postprocedure vision loss and possibility of the need for additional surgery. Thereupon, in the consultation, held with participation of ophthalmology and hematology specialists, the patient was deemed to have aggressive lymphoma of the choroid due to rapid progression of choroidal infiltrates in the evaluation of the patient’s fundus examination and macular OCT, performed 6 weeks after the initial admission, and treatment decision was made without tissue diagnosis. Methotrexate 2.5 g/m^2^ biweekly (4000 gr/2 weeks, 7 doses), weekly rituximab (375 mg/week, 6 weeks in total), and radiotherapy to the left eye and left testis were planned for the patient. Fundus, macula OCT, and orbital USG images after fourth dose of methotrexate treatment are shown in Figures 2(a), 2(b), and 2(c). After the completion of fourth dose of methotrexate treatment, external beam radiotherapy (EBRT) was applied to the left orbit of the patient. The vision rate improved to 0.05 in the patient’s left eye. The follow‐up of the patient, whose orbita EBRT has been completed, has a stable course and 3 more doses of methotrexate treatment and palliative testicular RT are being planned.

## 3. Discussion

DLBCNHL is a malignancy with very variable prognostic features in terms of nodal‐extranodal involvement, location of the involved area, molecular genetic features, and cells constituting the tumor microenvironment. Extranodal involvement is one of the most important negative prognostic markers [14]. It is the most common lymphoma variant of the CNS and testes. CNS and testis lymphomas can be seen together, as well as recurrence of testicular lymphoma in the CNS after treatment or in the testes after treatment of CNS [15].

Choroidal lymphoma, which is a variant of CNS lymphoma, is a lymphoma that should be evaluated separately due to the difficulties in diagnosis and the lack of a standard treatment protocol. Since it is a rare condition and can be confused with pathologies such as uveitis and retinopathy at the time of diagnosis, choroidal lymphoma requires a detailed and multidisciplinary clinical approach [16]. Inconclusive results can be obtained in half of tissue biopsy samples, especially in thin choroidal involvement, during histopathological diagnosis [8]. It may cause the patient to reject the invasive sampling procedure due to high vision‐related complications such as cataract, vitreous hemorrhage, and retinal detachment [17]. There is an urgent demand for a noninvasive, minimally invasive, or more safer diagnostic tool.

CNS and testis lymphomas have common phenotypic, genotypic, and biological features [18]. These regions are considered as IPS and require different treatment approaches due to this feature. Overactive NF‐kB pathway of lymphomas of this region, 9p24.1 aberration, and prevalence of programmed death ligand (PD‐L1)–expressing lymphocytes and macrophages in the tumor microenvironment are the main molecular and cellular features, distinguishing lymphomas of these regions from other lymphomas [19].

IPS lymphomas are mostly of nongerminal origin. They usually express MYC and BCL2 (double expressor). Loss of HLA expression leads to decreased MHC expression, as a result of which tumor cells get rid of cytotoxic T‐lymphocytes and other immune mechanisms by decreasing antigen presentation [20].

Although there is no generally accepted protocol for the treatment of indolent subtypes of choroidal lymphoma, rituximab, chemotherapy, immunotherapy, steroid, EBRT, and untreated follow‐up are the general approaches applied in clinics [14]. On the other hand, since systemic involvement is more common in aggressive lymphomas of the choroid, systemic chemotherapy is more frequently used. Although there is a limited amount of data in the literature based on case series or reports, a retrospective study by Mashayekhi and colleagues at a single center examined 59 cases of primary and secondary choroidal lymphoma. The study assessed the effects of treatments such as systemic steroids, intravitreal methotrexate, chemotherapy, and EBRT on these cases. Although systemic chemotherapy was not administered for primary choroidal lymphoma, the cumulative complete response rates were similar for both primary and secondary choroidal lymphoma. However, it was found that secondary choroidal lymphoma cases had higher mortality rates due to lymphoma and nonlymphoma causes [7]. In most of the literature cases, patients were treated after tissue diagnosis. However, due to the complication risk of invasive procedures for diagnosis, treatment can be performed without tissue diagnosis in special cases [14]. In a retrospective study conducted by Valenzuela and colleagues, covering cases from January 1951 to April 2019, it was highlighted that most patients with ocular diffuse large B‐cell lymphoma were diagnosed postmortem or following enucleation. The study also emphasized that patients diagnosed during this period had a shorter life expectancy [8]. Although there are studies stating that anthracycline‐based treatments increase the response in testicular lymphoma, as in our case, adding CNS‐targeted high‐dose systemic and intrathecal methotrexate to the treatment improves the results in preventing CNS recurrence [21].

In our case, the presentation of a patient with untreated testicular lymphoma with choroidal recurrence 18 months after the initial diagnosis was evaluated. The treatment for this region and possible systemic involvement was successfully applied and the patient was followed up clinically. Purely surgical treatment of IPS nonindolent lymphoma carries the risk of recurrence for the later periods and this condition requires systemic treatment, including in early disease stages, such as stage 1. Due to the inability of imaging, such as PET‐CT of lymphomas of these regions, to exclude micrometastases, it would be more appropriate to administer systemic treatment after diagnosis. Although IPS lymphoma is a very new concept, new treatment approaches need to be developed specifically for these cases.

## 4. Conclusion

In line with all these data, a separate standard treatment and diagnostic approach is needed for lymphomas of these regions. In addition, it must be taken into account that these regions may also have indolent lymphomas. Detailed examination by pathologists of indolent forms, where only surgical excision may be sufficient, and high‐grade lymphomas, where the possibility of aggressive recurrence may be high, is important in determining treatment approaches such as surgical excision and chemotherapy.

## Ethics Statement

This article does not contain any studies with human participants or animals performed by any of the authors. Ethical approval is not required for the retrospective analysis of this case.

## Consent

Written informed consent was obtained from the patient for publication of this case report.

## Disclosure

Consent for publication was given by all authors and necessary parties prior to submission.

## Conflicts of Interest

The authors declare no conflicts of interest.

## Author Contributions

Dr. Veysel Erol and Dr. Nevin Alayvaz Aslan prepared the manuscript by following up and treating the patient. Dr. Veysel Erol wrote the article with inputs from all authors.

Dr. Osman Parça made patient’s ophthalmological evaluation.

Dr. Nilay Şen Türk evaluated the pathological specimen.

## Funding

The authors received no specific funding for this work.

## Data Availability

Data sharing is not applicable to this article, as no new data were generated or analyzed in this case report.
